# Periodized Aerobic Interval Training Modifies Geometric Indices of Heart Rate Variability in Metabolic Syndrome

**DOI:** 10.3390/medicina55090532

**Published:** 2019-08-26

**Authors:** Laís Manata Vanzella, Denise Brugnoli Balbi Dagostinho, Maria Paula Ferreira de Figueiredo, Carlos Iván Mesa Castrillón, Jayme Netto Junior, Luiz Carlos Marques Vanderlei

**Affiliations:** 1 School of Technology and Sciences, São Paulo State University (UNESP), 19060-900 Presidente Prudente, Brazil; 2 Faculty of Health Sciences, The University of Sydney, 456P+HW Sydney, Australia

**Keywords:** metabolic syndrome x, exercise, autonomic nervous system, heart rate, heart rate variability

## Abstract

*Background:* Metabolic syndrome (MetS) influences the autonomic modulation, increasing the risk of cardiovascular events, which demands the identification of effective treatments for this population. Considering this, the study has the objective of evaluating the effects of periodized aerobic interval training (AIT) on geometrical methods of heart rate variability (HRV) on individuals with MetS. *Methods:* 52 individuals with MetS were considered for analysis. They were divided into two groups: aerobic interval training group (AITG; *n* = 26) and control group (CG; *n* = 26). The AITG performed 16 weeks of periodized AIT. For HRV analysis, the heart rate was recorded beat-by-beat at the beginning and the end of the AIT program and geometrical methods were used for analysis. *Results:* significant increase was observed for triangular index (RRtri, −1.25 ± 0.58 vs. 1.41 ± 0.57), standard deviation of distances from diagonal to points (SD1, −0.13 ± 1.52 vs. 4.34 ± 1.49), and standard deviation of distances from points to lines (SD2, −2.14 ± 3.59 vs. 11.23 ± 3.52) on AITG compared to CG. Significant differences were not observed for triangular interpolation of normal heartbeats interval histogram (TINN, −4.05 ± 17.38 vs. 25.52 ± 17.03) and SD1/SD2 ratio (0.03 ± 0.02 vs. 0.00 ± 0.02). Qualitative analysis of the Poincaré plot identified increase on dispersion of both short and long-term intervals between successive heartbeats (RR interval) on AITG after the AIT program. *Conclusion:* geometric indices of HRV suggest an increase in cardiac autonomic modulation in individuals with MetS after 16 weeks of periodized AIT.

## 1. Introduction

Heart rate variability (HRV) is a noninvasive measure used to identify phenomena related to the autonomic nervous system [1]. Different methods are used to analyze the HRV, as geometrical methods, that allow the presentation of intervals between successive heartbeats (RR intervals) in geometric patterns and the use of standard approximations on HRV measures. Triangular index (RRtri), triangular interpolation of normal heartbeats (NN) interval histogram (TINN), and Poincaré plot are indices extracted by geometrical methods [1,2,3].

Some characteristics could highlight geometrical methods as a form of HRV analysis. RRtri and TINN are highly insensitive to artifacts and ectopic beats, which reduces the necessity of pre data processing, disregarding values outside the curve for analysis [4]. Moreover, qualitative analysis of Poincaré plot provides a visual HRV analysis that facilitates the data interpretation. This type of analysis is a graphic representation considered by some authors as based on nonlinear dynamics [5,6,7,8] and mechanisms involved in cardiovascular regulation appear to interact with each other in a non-linear way [9,10].

Using geometrical methods, studies have demonstrated HRV decrease on individuals with metabolic syndrome (MetS), defined as a disorder represented by a cluster of at least three cardiovascular risk factors [11]. Individuals with MetS have HRV reduction compared to healthy subjects, suggesting a worsened cardiovascular regulation [12] and increase on cardiovascular risk events [13,14,15], arrhythmia and higher morbidity and mortality [15].

High-intensity aerobic interval training (AIT) is described as an important strategy to improve the autonomic modulation, which influences sympathetic-vagal balance and parasympathetic modulation increases in individuals with different conditions [16,17,18], such as MetS [19]. To our knowledge, however, there are no studies that report the effects of a periodized aerobic interval training (AIT) on geometric indices on this population.

Periodized trainings were created years ago as a strategy to increase the athletes’ performance [20]. However, this concept of training could be directed to different types of competition [21] and also as a strategy of treatment for specific conditions, as MetS. Considering this, it is important to understand if this type of treatment could be able to influence the geometric indices of HRV in sedentary adults with MetS, reducing the cardiovascular risks in this population. Furthermore, as cited above, Poincaré plot is considered as a nonlinear method of HRV, and it is suggested that cardiovascular regulation interacts nonlinearly [9,10], contributing to a better understanding of the cardiovascular risk of individuals with MetS.

Considering the data as a whole, some gaps are pointed in the literature. Could periodized AIT promote cardiac autonomic modulation changes, identified by geometric indices of HRV? If so, which changes will occur? This study design intends to answer these questions.

In this sense, the objective of the study is to evaluate the effects of a periodized AIT on geometric indices of HRV on individuals with MetS. We hypothesized that geometrical methods of HRV will allow us to identify positive changes on cardiac autonomic modulation of individuals with MetS, increasing parasympathetic and global modulation.

## 2. Materials and Methods

### 2.1. Study Nature and Casuistry

The study is characterized as a non-randomized clinical trial that evaluates the effects of a periodized AIT on geometric indices of HRV on individuals with MetS. This clinical trial was prospectively registered in ClinicalTrials.gov (NCT03119493).

For recruitment, information about the study population and characteristics were disseminated through campaigns in public squares and digital media (television, websites and radio) and flyers distributed at supermarkets, financial institutions, pharmacies, health posts and fire brigade.

Individuals with MetS were included in the study. MetS was defined according to International Diabetes Federation (IDF) [11], considering at least three out of five cardiovascular risk factors, as follows: glucose ≥100 mg/dL or treatment for hyperglycemia; High-density lipoprotein (HDL) cholesterol <40 mg/dL for men and <50 mg/dL for women or treatment for low HDL; Triglycerides ≥150 mg/dL or treatment for higher triglycerides; obesity: waist circumference ≥90 cm for men or ≥80 cm for women; hypertension: blood pressure ≥130 × 85 mmHg or treatment for hypertension [11].

Individuals who: (1) performed regular physical activity six months previous to data collection; (2) had muscle-tendon or osteoarticular injury in lower limbs and/or spine; (3) had respiratory, neurological or cardiovascular disease; (4) were alcoholics and/or smokers were not included Furthermore, subjects who changed their medication during the study were excluded. 

The sample size was calculated according to Massaro & Pecchia [16], which suggests a minimum of 40 subjects for studies considering HRV analysis. Thus, 30% of this value was increased, considering possible sample loses. The calculation of the study power with the number of subjects analyzed and significance level of 5% (two-tailed test) confirmed a power higher than 80% to detect differences between the variables.

### 2.2. Ethics Criteria

The procedures of the study were approved by the Committee for Ethics and Research of the institution (CAAE: 53117116.0.0000.5402; Date: March 2016). The volunteers were informed of the procedures and objectives of the study, and after agreeing to participate, signed a consent form. In addition, each volunteer attached a copy of a medical certificate provided by a Cardiologist, confirming them to be in a sufficient physical condition to perform the exercises.

### 2.3. Experimental Approach

Initially, an interview was performed to identify personal data (name, sex and age) and medicines used (self-reporting during all study). MetS diagnosis was confirmed using glycemia, triglycerides and HDL-cholesterol exams as well as blood pressure and waist circumference measures and/or considering the use of medicines for MetS treatment. Anthropometric data (weight, height, abdomen circumference and waist/hip circumference) were evaluated for sample characterization. Then, the heart rate (HR) was recorded beat-by-beat for 30 min, at rest, in supine position, using a heart rate monitor (Polar S810i, Kempele, Finland) to further HRV analysis.

After the initial assessments, the volunteers with MetS were allocated by convenience into two groups: periodized AIT group (AITG) and control group (CG) and instructed to maintain their diet and daily habitual activity during the study. The AITG performed the periodized AIT three times per week with 24 to 72 h of interval, for 16 weeks totalizing 39 sessions. The CG did not perform any treatment for 16 weeks. 

Seven days after the end of the study protocol, the HR was recorded beat-by-beat in both groups, for further HRV analysis. At AITG, subjects with at least 85% of presence were considered for analysis and an intention to treat was carried out for the remaining volunteers. 

### 2.4. Training Program

Periodized AIT was performed on a treadmill (Movement, Professional LX-160, Pompéia, Brazil and Inbramed, Export, Porto Alegre, Brazil), for 16 weeks (39 sessions), three times per week with interval of 24 to 72 h, 30 to 75 min per session. 

Each session of the periodized AIT was divided into three phases: (1) warm up: five minutes of upper and lower limbs global stretching and five minutes of walking with HR less than 20% of heart rate reserve (HRR); (2) training phase, performed progressively, with intensity of 19 to 90% of HRR [22] and active recovery between the series with 19 to 50% of HRR, according to the respective training phase (light, moderate, and high) (Table 1); and (3) cool down: five minutes of walking with HR less than 20% of HRR and five minutes at rest.

As it can be observed in Table 1, the periodized AIT was divided according to the intensity levels, light (1) (intensity of 20–39% of HRR and recovery of 19% of HRR), moderate (2) (intensity of 40–59% of HRR and recovery of 30% of HRR), and high (3) (intensity of 60–90% of HRR and recovery of 50% of HRR).

The intensity, number of series, and effort time were fixed for all AITG volunteers, while recovery interval between series (1 to 4 min), total time (sum of total effort time and recovery time) and speed were established individually considering the HR response during the session and the intensity level (light, moderate or high).

The intensity of the AIT and the HR recovery was determined though HRR [19] according to the formula: HRR = (HR_max_ − HR_rest_) × % training + HR_rest_. The maximal HR (HR_max_) was obtained through the Karvonen formula (HR_max_ = 220 − age (in years)) [22,23]. Resting HR (HR_rest_) was obtained using a HR monitor with the volunteers at rest for 30 min, considering the HR mean recorded from 5 to 25 min.

If the volunteers used beta-blocker, the HR was corrected according to the formula: HR Correction = *y* + 95.58/9.74 [21], where *y* is the dose in mg of propranolol or equivalent drug (Kaplan table) [24]. The percentage resulting from this formula was subtracted from the HR_max_ for further corrections to the HRR.

To increase the safety, blood pressure (BP) and HR of the volunteers were measured each session (before the warm-up, at the beginning of each active recovery, and after the cool-down), possible cardiovascular signs (pulse rate alterations, systolic blood pressure >200 mmHg, diastolic blood pressure >120 mmHg, tachypnea and pallor) and symptoms (fatigue, muscle pain, angina, dizziness, nausea and cramp) were monitored during the sessions and Borg scale was applied at the end of the cool-down to verify the perceived effort during the training.

### 2.5. Heart Rate Variability Evaluation

Cardiac autonomic modulation was evaluated at the beginning and the end of the study protocol (16 weeks) through geometrical methods of HRV, during the morning period (7:00 to 11:00 a.m.) in an artificially heated room from 21 °C to 24 °C and humidity from 40 to 60%. The volunteers were instructed not to consume stimulating substances such as tea, coffee, soda, chocolate, and alcohol for 24 h prior to the HRV analysis.

To HRV analysis, the HR was recorded beat-by-beat for 30 min, with volunteers at rest, in supine position, without moving or talking and breathing spontaneously. Circulation of people in the room was not permitted during the HR record, to reduce the anxiety and to avoid capture errors.

In order to evaluate HRV, data on the interval between heart beats (RR intervals) were sent to a microcomputer, by the pulse receptor’s data transmission port to Polar ProTrainer 5 software (5.41.002 version, Kempele, Finland), using an infrared signal interface. Only series with less than 5% error were included in the study. The RR intervals series passed initially by filtering through standard filter Polar Precision Performance software (Polar Electro, Finland [25,26]).

Geometrical methods, used to HRV analysis, convert the RR intervals in geometrical patterns [1,2,3]. Triangular index (RRtri), triangular interpolation of NN interval histogram (TINN), and Poincaré plot (SD1, SD2, and SD1/SD2 ratio) are indices extracted by this method.

The RRtri index was calculated from the construction of the histogram density of normal RR intervals; the horizontal axis (*x* axis) which contains all the possible RR values and vertical axis (*y* axis), the frequency of occurrence. From the tips of the histogram columns, we obtained a figure similar to a triangle [1]. The TINN is the baseline distribution measured as a triangular base, approximating the distribution of all RR intervals. The difference of least squares was used to determine the triangle [1].

The Poincaré plot is a map of points in Cartesian coordinates, constructed from RR values, where each point is represented on the *x*-axis by the previous RR interval and on the *y*-axis by the following interval, forming a figure that allows quantitative and qualitative analysis [27]. On quantitative analysis, SD1 (standard deviation of distances from diagonal to points), SD2 (standard deviation of distances from points to lines) [27], and SD1/SD2 ratio (ratio between short and long RR intervals variations) were calculated [27], while qualitative analysis of the Poincaré plot is a visual analysis of the figure formed by the attractor: (a) normal plot: high RR interval dispersion and (b) lower global dispersion beat-by-beat without long-term RR intervals [15].

Kubios HRV Analysis software version 2.0 was used to calculate these indices (University of Eastern Finland, Kupio, Finland) [13].

### 2.6. MetS Diagnosis

To confirm MetS diagnosis [1], blood samples of the volunteers were collected by the Laboratório de Análises Clínicas-UNILAB (UNILAB, Presidente Prudente, São Paulo, Brazil) and automated dry chemical analysis was used to determine values of glycaemia, triglycerides and HDL-Cholesterol. Obesity was determined according to waist circumference values, measured according to Brazilian Guidelines on Obesity [28] and hypertension was determined indirectly with a sphygmomanometer and stethoscope according to the VI Brazilian Guidelines on Hypertension [29]. It was also considered for MetS diagnosis the use of medicines for MetS treatment. 

### 2.7. Data Analysis

Descriptive statistics were used to describe the population profile, with results presented as mean, standard deviation, median, minimum, maximum, and confidence interval of 95%. Chi-squared test was used to analyze differences between MetS components and Class of medicines.

Differences between the HRV indices obtained at the end and the start of the training protocol were used to compare CG and AITG. Analysis of Covariance (ANCOVA) was used for analysis. It allows the comparison of one variable in 2 groups taking into account (or to correct for) variability or other variables, called covariates (sex, age and high blood pressure). Significance level was fixed at 5%. SPSS version 13.0 (University of Chicago, Chicago, IL, USA) was used for the analysis.

## 3. Results

**Figure 1:**
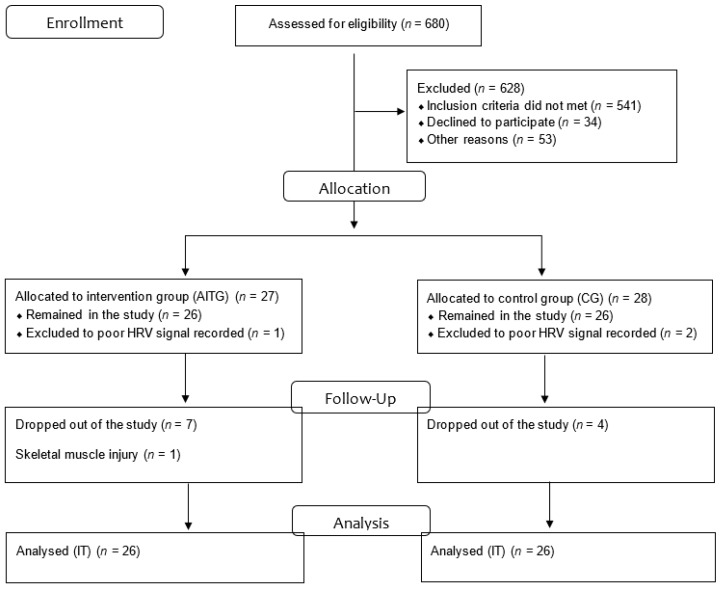
Flow gram; IT: Intention to treat.

In the study, 680 individuals were assessed for eligibility and 628 were excluded (541 did not meet the inclusion criteria, 34 declined to participate, and 53 declined for other reasons). Thus, 55 subjects with MetS diagnosis (confirmed as item 2.6), of both genders, aged 40–60 years, participated effectively in the study. After the allocation, at the aerobic interval training group (AITG), 1 volunteer was excluded to poor HRV signal record, 1 to a skeletal muscle injury unrelated to the training protocol, and 7 dropped out of the study. At the control group (CG), 2 volunteers were excluded to poor HRV signal record and 4 dropped out for personal reasons (Figure 1)

Table 2 describes the characterization, Metabolic syndrome (MetS) components and class of medicines used, separated according to groups (aerobic interval training group, AITG and control group, CG). Significant differences were observed only for weight. 

The effects of periodized AIT on geometric indices of HRV are described on Table 3. Significant increase on RRtri, SD1, and SD2 was observed for AITG (*p* < 0.05). On the other hand, TINN and SD1/SD2 ratio significant differences were not observed.

**Figure 2:**
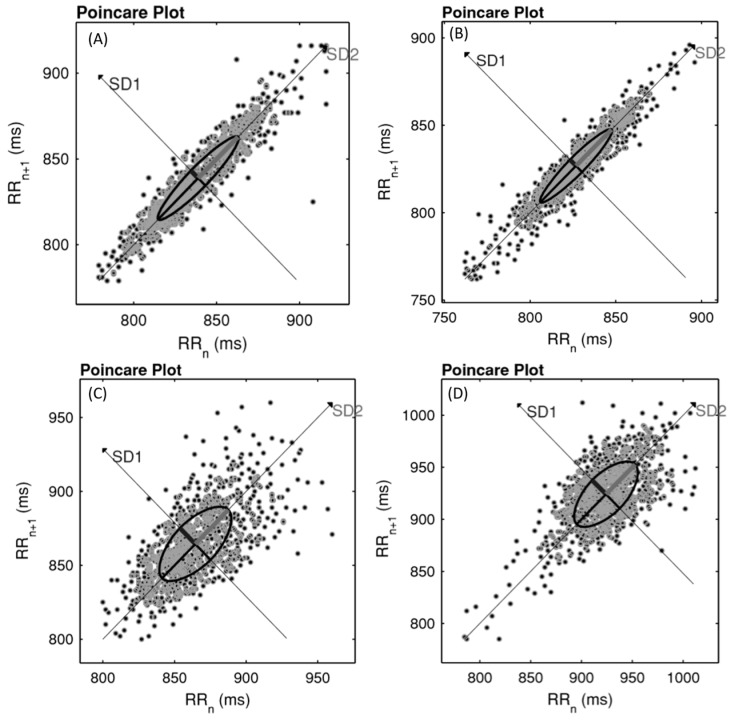
Example of Poincaré Plot observed in control group (CG) (pre (**A**; SD1 = 6.2; SD2 = 34.0) and post (**B**; SD1 = 5.0; SD2 = 29.4)) and aerobic interval training group (AITG) (pre (**C**; SD1 = 15.1; SD2 = 32.7) and post (**D**; SD1 = 19.2; SD2 = 40.7)).

Qualitative analysis of Poincaré Plot can be observed on Figure 2, at pre and post-training moments for both groups (CG and AITG). Subjects were selected according to mean values of SD1 and SD2. 

## 4. Discussion

The main results of the study demonstrate a significant increase on RRtri, SD1 and SD2 for AITG, compared to CG. Additionally, qualitative analysis of Poincaré Plot indicates increase on short-term and long-term. These results can suggest parasympathetic and global modulation increase on individuals with MetS diagnosis after 16 weeks of periodized AIT. 

This is the first study to evaluate the effects of a periodized AIT on geometric indices of HRV on individuals with MetS. These indices allowed for the identification of modifications in cardiac autonomic modulation induced by periodized AIT, characterized as increases in global variability (RRtri and SD2) and parasympathetic modulation (SD1). Improvement in cardiac autonomic modulation was also observed through qualitative analysis of Poincaré plot (RR interval dispersion beat-by-beat increase on short-term and long-term at AITG after training).

Positive effects of AIT have been demonstrated in different populations using linear indices of HRV on time and frequency domain [17,30]. Munk et al. [30] and Rakobowchuk et al. [17] reported cardiac autonomic modulation improvement after high-intensity AIT performed by individuals after percutaneous coronary intervention and healthy subjects, respectively. 

According to Ramos et al. [19], high-intensity AIT also promotes improvement on cardiac autonomic modulation of individuals with MetS, identified using linear methods and quantitative analysis of Poincaré plot (SD1 and SD2) [19]. Our study was performed using a periodized AIT with progressive increase of intensity (light, mild and high), identifying results similar to Ramos et al. [19]. 

Even though positive effects of high- intensity AIT are reported on individuals with MetS using quantitative analysis of Poincaré plot [19], the use of RRtri, TINN and qualitative analysis of Poincaré plot are not described. As reported above, RRtri and TINN are highly insensitive to artifacts and ectopic beats, which reduces the necessity of pre data processing, disregarding values outside the curve [4]. Additionally, qualitative analysis of Poincaré plot provides a visual HRV analysis that facilitates the data interpretation. This type of analysis has been considered as a nonlinear method of HRV [9,31,32] and mechanisms involved in cardiovascular regulation appear to interact with each other in a non-linear way [9,10] enabling important information about cardiovascular risk of individuals with MetS.

The increase on cardiac autonomic modulation observed in this study could be related to angiotensin II reduction and nitric oxide increase, which appears to be directly related to the vagal modulation increase as a chronic effect of the exercise training [33,34]. Individuals with MetS have a cardiac autonomic modulation reduction compared to healthy subjects, and consequently a worsened cardiovascular regulation [12] and an increase of cardiovascular risk events [14,15], arrhythmia and higher morbidity and mortality [15]. 

No significant difference was observed for SD1/SD2, which can be justified by the significant increase on SD1 and SD2 on AITG. For TINN, an increase was observed after periodized AIT, but with no differences. TINN is determined by the width of the distribution measured as a base of a triangle, approximating the NN interval distribution [1]. With this approximation, discrepant NN intervals directly influence the indices, which could contribute to the absence of significance related to this variable. No difference was also observed forMeanRR and short variations of this indices could justify this result, however, a reduction on HR could be observed for AITG (Mean (SE) = −1.03 (1.81)), suggesting a vagal modulation increase. 

This study has limitations related to the randomization of the volunteers. The periodized AIT was scheduled in the evening, impairing the adherence of volunteers that did not have transport and live far away from the place of the training. These volunteers were allocated to CG. 

Despite this, some aspects reinforce our findings. Variables that could influence cardiovascular and autonomic responses such as sex, age, and medicines for high blood pressure treatment were considered as confounding factors during the statistical analysis process and an intention to treat analysis was realized in individuals that did not complete the periodized AIT. Furthermore, the study suggests that 16 weeks of periodized AIT improve the HRV and consequently the cardiovascular regulation by the autonomic nervous system, reducing cardiovascular risk of individuals with MetS. 

For future researches, further investigations considering HRV assessment on each phase of the periodized AIT, performed by individuals with MetS, are encouraged. 

## 5. Conclusions

We concluded that geometric indices of HRV suggest an increase on cardiac autonomic modulation in individuals with MetS after 16 weeks of periodized AIT.

## Figures and Tables

**Table 1:** Periodized aerobic interval training (AIT) program: number of sets and repetitions, effort time, recovery time, total time, and intensity.

Week (Phase)	Sessions	Sets × Effort Time	Recovery Time Between Sets	Total Time (Effort Time + Recovery Time)	Intensity
1st	1st and 2nd	5 × 4 min	1–4 min	24–32 min	Training: 20–39% of HRR Recovery: 19% of HRR (Light)
2nd	3rd and 4th	6 × 4 min	1–4 min	29–39 min	
3rd	5th and 6th	7 × 4 min	1–4 min	34–46 min	
4th	7th, 8th and 9th	8 × 4 min	1–4 min	39–53 min	
5th	10th, 11th and 12th	9 × 4 min	1–4 min	44–60 min	
6th	5th Recovery week				
(Adaptation)	13th, 14th and 15th	9 × 4 min	1–4 min	44–60 min	
7th	16th and 17th	4 × 2.5 min	1–4 min	13–19 min	Training: 40–59% of HRR Recovery: 30% of HRR (Moderate)
8th	18th and 19th	5 × 2.5 min	1–4 min	16.5–24.5 min	
9th	20th and 21st	6 × 2.5 min	1–4 min	20–30 min	
10th	22nd, 23th and 24th	7 × 2.5 min	1–4 min	23.5–41 min	
11th	25th, 26th and 27th	7 × 2.5 min	1–4 min	23.5–41 min	
(Intermediate)	11th Recovery week				
12th	28th and 29th	5 × 1.5 min	1–4 min	11.5–19.5 min	Training: 60–90% of HRR Recovery: 50% of HRR (High)
13th	30th and 31th	6 × 1.5 min	1–4 min	14–24 min	
14th	32nd and 33th	7 × 1.5 min	1–4 min	16.5–28.5 min	
15th	14th Recovery week				
16th	34th, 35th and 36th	8 × 1.5 min	1–4 min	19–33 min	
(Final)	37th, 38th e 39th	9 × 1.5 min	1–4 min	21.5–37.5 min	

HRR: heart rate reserve.

**Table 2:** Characterization, MetS components, and class of medication used by the volunteers separated according to groups, AITG and CG.

	AITG (*n* = 26)	CG (*n* = 26)	*p* Value
Age (years)	49.96 ± 6.53 (40.00–59.00)	52.44 ± 6.42 (40.00–60.00)	0.16
Waist-hip ratio	0.93 ± 0.05 (0.83–1.04)	0.91 ± 0.06 (0.72–1.04)	0.23
AC (cm)	111.67 ± 10.39 (96.00–133.00)	107.61 ± 9.96 (94.50–133.00)	0.15
Weight (kg)	95.11 ± 16.39 (64.40–127.10)	83.25 ± 16.89 (56.00–124.7)	0.01
Height (m)	1.71 ± 0.09 (1.52–1.91)	1.63 ± 0.08 (1.47–1.80)	0.07
BMI (kg/m^2^)	32.38 ± 4.28 (23.94–40.56)	30.75 ± 4.42 (23.92–39.59)	0.18
MetS components (%)			
BP	80.76	80.76	1.00
Blood glucose	69.23	69.23	1.00
Triglycerides	73.07	38.46	0.08
Low HDL	57.69	46.15	0.57
WC increased	100.00	100.00	1.00
Class of medicines (%)			
Ca^+^ channel blocker	11.53	19.23	0.70
Antagonist of Angiotensina II	46.15	34.61	0.39
Thiazide diuretics	30.76	34.61	1.00
Beta blockers	19.23	42.30	0.13
ECA inhibitors	15.38	7.69	0.66
Insulin	7.69	23.07	0.24
Sulfonylurea	3.84	26.92	0.05
DPP4inhibitor	3.84	3.84	1.00
Metformin	19.23	30.76	0.52
Statin	26.92	30.76	1.00
Fibrate	7.69	0.00	0.49
Thiazolidinedione	0.00	7.69	0.49
Antiplatelet agent	7.69	3.84	1.00
Others	34.61	23.07	0.54

Average ± standard deviation (minimum–maximum). Legends: AC = abdominal circumference; BMI = body mass index; MetS = Metabolic syndrome; cm = centimeters; kg = kilogram; m = meters; m^2^ = square meters; % = percent; HBP = high blood pressure; HDL = high density lipoprotein; Ca^+^ = Calcium; ECA = angiotensin converting enzyme; DPP4 = Dipeptidyl peptidase-4; HDL = high density lipoprotein.

**Table 3:** Comparison of deltas of HRV geometric indices adjusted by sex, age and presence of medication for blood pressure control of AITG and CG group.

	CG	AITG				
Variables	Mean (SE)	Mean (SE)	F	P	Eta squared	Effect size
RRTRI	−1.25 (0.58)	1.41 (0.57)	10.10	0.00	0.18	High
TINN	−4.05 (17.38)	25.52 (17.03)	1.40	0.24	0.03	Low
SD1	−0.13 (1.52)	4.34 (1.49)	4.20	0.04	0.08	Moderate
SD2	−2.14 (3.59)	11.23 (3.52)	6.71	0.01	0.12	High
SD1/SD2	0.03 (0.02)	0.00 (0.02)	0.66	0.41	0.01	Low
Mean RR	−14.10 (31.59)	−0.53 (30.94)	0.08	0.76	0.00	Low

Legend: AITG = aerobic interval training group; CG = control group; SE = standard error; RRTRI = triangular index; TINN = triangular interpolation of NN interval histogram; SD1 = standard deviation of distances of diagonal points; SD2 = standard deviation of distances from points to lines.
